# Clinical Impact of Empiric Ceftriaxone for Hospitalized Patients with Community-Onset Healthcare-Associated UTIs

**DOI:** 10.3390/jcm14248761

**Published:** 2025-12-11

**Authors:** Manuel Madrazo, Ian López-Cruz, Laura Piles, María Civera, José María Eiros, Juan Alberola, Arturo Artero

**Affiliations:** 1 Doctor Peset University Hospital, Universitat de València, Avda Gaspar Aguilar n 90, 46017 Valencia, Spain; manel.madrazo@gmail.com (M.M.); ilopezcruz5@gmail.com (I.L.-C.); laurapilesroger@gmail.com (L.P.); civeramariabar@gva.es (M.C.); arturo.artero@uv.es (A.A.); 2 Rio Hortega University Hospital, Universidad de Valladolid, 47014 Valladolid, Spain; eiros@med.uva.es

**Keywords:** ceftriaxone, UTI, healthcare-associated infections, inappropriate empirical antibiotic therapy

## Abstract

**Background/Objectives**: Ceftriaxone is widely used as empirical antimicrobial therapy (EAT) for urinary tract infections (UTIs). However, healthcare-associated urinary tract infections (HCA-UTIs) are often associated with inadequate EAT (IEAT). This study aims to evaluate the clinical impact of ceftriaxone as EAT in patients admitted to the hospital with community-onset HCA-UTIs in a setting with relatively high rates of antimicrobial resistance. **Methods**: A prospective observational study was conducted, comparing patients who received empirical treatment with ceftriaxone to those treated with other antibiotics. **Results**: A total of 235 cases were analyzed, 50.2% received ceftriaxone as EAT. The median age was 79 years, and 47.2% of patients were female. IEAT was significantly more frequent in the ceftriaxone group (36.4% vs. 17.1%, *p* = 0.001). Thirty-day mortality was 11.1%, with no significant difference between the ceftriaxone and non-ceftriaxone groups (11.9% vs. 10.3%, *p* = 0.752) but the use of antibiotics other than ceftriaxone was associated with a longer hospital stay (6 [4–8] vs. 5 [3–7] days, *p* = 0.037). The use of ceftriaxone as EAT was not associated with an increased risk of recurrence (16.1% vs. 15.4%, *p* = 0.709). **Conclusions**: In summary, empirical ceftriaxone use in patients with community-onset HCA-UTI was associated with a higher rate of inappropriate empirical therapy; however, it did not increase mortality or recurrence and was associated with a shorter hospital stay. These findings support the use of ceftriaxone as a potential option in selected patients without septic shock, while highlighting the importance of considering local resistance patterns and individual patient risk factors.

## 1. Introduction

Urinary tract infections (UTIs) are among the most common bacterial infections worldwide, second only to respiratory tract infections as a cause of hospitalization [1]. Healthcare-associated urinary tract infections (HCA-UTIs) represent a significant burden, contributing to increased morbidity, antimicrobial resistance, prolonged hospital stays, and higher healthcare costs [2]. In particular, community-onset HCA-UTIs are increasingly recognized among patients admitted with infectious diseases due to their clinical impact and management challenges [2].

Ceftriaxone, a third-generation cephalosporin, is widely employed as empirical antibiotic therapy (EAT) for UTIs because of its broad spectrum of activity against the most frequent uropathogens, including *Escherichia coli*, *Klebsiella pneumoniae*, and *Proteus mirabilis* [3].

However, in HCA-UTIs, the prevalence of extended-spectrum β-lactamase-producing Enterobacterales (ESBL-E) and other pathogens inherently resistant to ceftriaxone, such as *Enterococcus faecalis* and *Pseudomonas aeruginosa*, is considerably higher. A multicenter prospective study in Spain [4] reported that 61.2% of healthcare-associated bacteremic UTIs exhibited a multidrug-resistant profile, with ESBL-E accounting for 25% of cases. Similarly, an observational study of hospitalized patients with complicated UTIs in the United States [5] found substantial resistance to commonly used antimicrobials, including third-generation cephalosporins and fluoroquinolones; notably, up to 13% of isolates were triple-resistant, a feature associated with higher rates of inappropriate empirical antibiotic therapy (IEAT), length of hospital stay, and increased costs.

Furthermore, a Spanish study of community-acquired complicated UTIs in older adults reported *E. faecalis* in 13.2% of cases [6], while other European and North American studies found that *P. aeruginosa* accounted for 4% to 7.7% of HCA-UTIs [5,7,8]. These pathogen distributions contribute to reported IEAT rates of 18.9% to 21% in HCA-UTIs [4,8], which have been linked to increased length of hospital stay and higher mortality.

In this context, the present study aims to assess the clinical impact of ceftriaxone as empirical antibiotic therapy in patients admitted with community-onset HCA-UTI, given the growing concern about antimicrobial resistance in this setting.

## 2. Material and Methods

**Figure 1:**
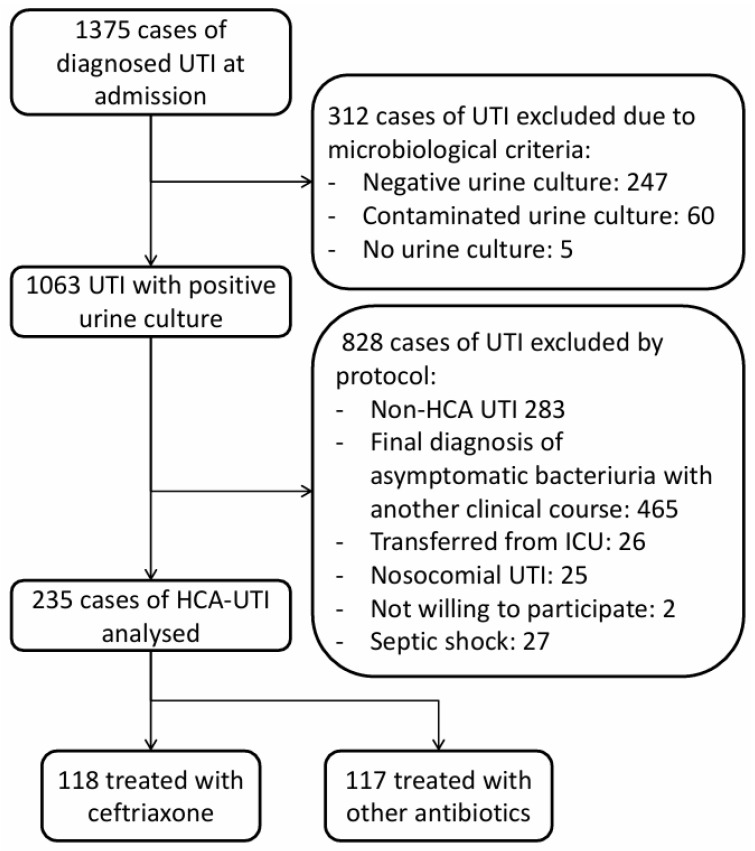
Flowchart of the inclusion process for 235 cases of community-onset healthcare associated urinary tract infection.

A prospective observational study was conducted at a university hospital between January 2017 and December 2022, enrolling patients admitted with a diagnosis of community-onset UTI who met the criteria for healthcare-associated infection as defined by Friedman et al. [9] The initial diagnosis was established by the attending physician in the emergency department and subsequently confirmed after admission by the responsible ward physician. Confirmation required a detailed review of the patient’s medical history, a comprehensive physical examination, and the application of laboratory and microbiological tests. Exclusion criteria included clinical presentations ultimately attributable to other conditions, nosocomial infections, UTIs acquired in the intensive care unit, absence of a positive urine culture, or refusal to participate (Figure 1). Patients in the ceftriaxone group received ceftriaxone monotherapy. Informed consent was obtained from all participants before inclusion. Each case was independently reviewed by two investigators prior to final enrollment. Epidemiological and clinical data were collected according to a pre-established protocol [10]. The study was approved by the Clinical Research Ethics Committee of Doctor Peset University Hospital (code 85/16 September 2016) and conducted in accordance with the STROBE statement.

UTI was defined as the presence of clinical symptoms (dysuria, hematuria, abdominal or costovertebral pain, urgency, or difficulty urinating), and a positive culture required at least 10^5^ colony-forming units per milliliter of urine [11]. HCA-UTI was defined as a UTI meeting any of the healthcare-associated infection criteria proposed by Friedman et al. [9]: (i) hospitalization in an acute care facility for ≥48 h within the previous 90 days; (ii) receipt of antimicrobial therapy within the previous 90 days; or (iii) residence in a nursing home. Recurrence was defined as a new episode of complicated UTI requiring hospital admission within 30 days after discharge.

Severity was assessed using the SOFA and qSOFA scores, measured within 24 h of admission to the Emergency Department (ED), and applied according to their original definitions [12]. The Charlson Comorbidity Index [13] was also calculated, with a score ≥ 3 considered as significant comorbidity. Sepsis was defined according to Sepsis-3 criteria, being sepsis an acute increase of ≥2 points in the total SOFA score attributable to infection [12]. The Acute Physiology and Chronic Health Evaluation II (APACHE II) score was used to assess illness severity at admission [14], and functional status was evaluated using the Barthel Index, which quantifies independence in activities of daily living.

Epidemiological and clinical variables were obtained from electronic health records, including age, sex, diabetes mellitus, cognitive impairment, chronic kidney disease (defined by an estimated glomerular filtration rate < 60 mL/min according to the CKD-EPI equation), chronic obstructive pulmonary disease, active or previous cancer, and presence of a chronic indwelling urinary catheter (defined as catheterization for >2 months). Prior antibiotic exposure was also recorded, as electronic prescription is mandatory in our region.

Clinical manifestations were documented through direct patient interviews and systematic physical examinations. Fever was defined as a body temperature ≥ 38 °C, either self-reported at home or confirmed in the ED. Laboratory tests included coagulation studies, complete blood counts, and comprehensive biochemical panels assessing hepatic and renal function, electrolyte concentrations, procalcitonin, and C-reactive protein.

Microbiological data were obtained from urine cultures and, when clinically indicated, blood cultures, together with antimicrobial susceptibility testing. This included identification of the causative pathogens of UTI, assessment of antimicrobial resistance profiles, detection of bacteremia, and recognition of polymicrobial infections. Urine culture was considered positive with the isolation of 1 or 2 pathogens, or 3 pathogens if the sample was collected using a sterile procedure, in a quantitative count ≥ 10^5^ CFU/mL. Urine culture was considered contaminated with the isolation of ≥3 pathogens if the sample was not collected using a sterile procedure or <10^5^ CFU/mL in a quantitative count. Urine culture isolates were identified using the Bruker MALDI Biotyper system (Beckman Coulter, Brea, CA, USA), and antimicrobial susceptibility testing was performed with the DxM MicroScan WalkAway system (Beckman Coulter, Brea, CA, USA), a microbroth dilution method following combined CLSI and EUCAST guidelines. Blood cultures were collected in the emergency department and processed with the BacT/ALERT^®^ VIRTUO™ automated system (bioMérieux Inc., Durham, NC, USA) for microbial growth detection. Microorganisms isolated from positive blood cultures were identified with the Bruker MALDI Biotyper system, and susceptibility testing was carried out using both the DxM MicroScan WalkAway (Beckman Coulter Inc., Carlsbad, CA, USA) and the VITEK 2 system (bioMérieux Inc., Durham, NC, USA), based on microbroth dilution methods in accordance with combined CLSI and EUCAST criteria.

Inadequate empirical antimicrobial therapy (IEAT) was defined as the failure to provide effective treatment once the causative pathogen and its antimicrobial susceptibility profile were known [15]. Multidrug-resistant bacteria (MDRB) were classified according to the international consensus by Magiorakos et al. [16] as non-susceptibility to at least one agent in three or more antimicrobial classes. For Gram-negative organisms, these included extended-spectrum penicillins, carbapenems, cephalosporins, aminoglycosides, and fluoroquinolones; for Gram-positive organisms, ampicillin, vancomycin, fluoroquinolones, fosfomycin, and linezolid. Extended-spectrum β-lactamase (ESBL)-producing Enterobacterales were initially suspected in isolates with reduced susceptibility to cefpodoxime, ceftazidime, cefotaxime, ceftriaxone, or aztreonam, and subsequently confirmed using the Double Disc Synergy Test (DDST), according to Clinical and Laboratory Standards Institute (CLSI) guidelines. ESBL production was defined by an enhanced inhibitory effect of a third-generation cephalosporin disc in the presence of clavulanic acid. A phenotypic confirmatory test was also performed for suspected AmpC β-lactamase-producing isolates, characterized by resistance to third-generation cephalosporins, a negative ESBL confirmatory test, or intermediate/resistant profiles to both amoxicillin–clavulanic acid and third-generation cephalosporins. Antimicrobial therapy modifications within the first 24–48 h of admission were retrieved from electronic medical records, whether prompted by clinical evolution or by adjustment to susceptibility testing results.

Continuous variables were summarized as medians with interquartile ranges (IQR), and categorical variables as absolute frequencies and percentages. The assumption of normality for quantitative variables was assessed using the Kolmogorov–Smirnov test. For normally distributed continuous variables, comparisons between two groups were performed with Student’s t test, and among more than two groups with analysis of variance (ANOVA). For non-normally distributed variables, the Mann–Whitney U test was applied. Categorical variables were compared using the chi-square test or Fisher’s exact test when expected frequencies were <5. The Kaplan–Meier method was used to compare survival rates between patients with and without sepsis. Cox proportional hazards regression was used to evaluate the significance of the observed differences between the survival curves. A two-tailed *p* value < 0.05 was considered statistically significant. Multivariate analysis was performed using logistic or negative-binomial regression, considering an α significance level of 0.05 for all tests. All analyses were conducted with IBM SPSS Statistics for Windows, version 22.0 (IBM Corp., Armonk, NY, USA). The sample size estimate was calculated a priori using previously published data [10]. Using a one-sided test, and assuming a 30-day mortality of 11.8%, a sample size of 259 patients was needed to achieve 80% power with 0.05 alpha error.

## 3. Results

During the study period, 1375 patients with UTIs were admitted to our medical ward. Of these, 235 cases of HCA-UTI were analyzed (Figure 1), and 118 patients (50.2%) received empirical therapy with ceftriaxone. In the non-ceftriaxone group, the most frequently used agents were meropenem (55 cases, 47%), followed by ertapenem (14 cases, 12%), piperacillin–tazobactam (5 cases, 4.3%), fosfomycin, and aztreonam (4 cases each, 3.4%).

The median age of the patients was 79 years, and 47.2% were female. Most patients had a high comorbidity burden (92.3% with a Charlson Index ≥ 3), with no significant differences between the ceftriaxone and non-ceftriaxone groups, except for dementia, which was more frequent in the ceftriaxone group (40.7% vs. 26.5%, *p* = 0.021) (Table 1).

Sepsis was present in nearly half of the patients (35.7%) at admission. The median APACHE II score was 11 [IQR 8–15]. No differences in severity at admission were observed between the groups, nor were there statistically significant differences in the presentation as acute pyelonephritis.

Bacteremia, presence of polymicrobial UTI, or multidrug-resistant bacteria showed no differences in both groups (Table 1). *Escherichia coli* was the most frequently isolated pathogen (52.6% of the cases), followed by *Klebsiella pneumoniae* (14.2%), *Pseudomonas aeruginosa* (9.1%), and *Enterococcus faecalis* (7.9%) (Table 2). Among patients receiving empiric ceftriaxone, *E. coli* was more commonly isolated compared with those treated with other regimens (56.5% vs. 48.8%), although the difference was not statistically significant (*p* = 0.277). *P. aeruginosa* also showed a tendency to the non-ceftriaxone group (6.5% vs. 11.6%, *p* = 0.225), but the difference was not significative. The distribution of other Gram-negative and Gram-positive microorganisms also did not significantly differ between groups.

Overall resistance rates are presented in Table 3. Among all isolates, resistance was highest to ampicillin (78.7%), ciprofloxacin (46.6%), and trimethoprim–sulfamethoxazole (44.1%). Resistance to third-generation cephalosporins was observed in 29.8% of isolates for ceftriaxone and 22.9% for ceftazidime. Carbapenem resistance remained uncommon (<3%). No significant differences in simple resistance patterns were observed between the groups, nor were observed in MDRB or BLEE-E (*p* 0.171 and 0.389, OR 0.84 (95% CI 0.64–1.09) and OR 0.86 (95% CI 0.60–1.23), respectively).

IEAT was significantly more frequent in the ceftriaxone group (36.4% vs. 17.1%, *p* = 0.001, OR 1.52 (95% CI 1.23–1.92)). In this group, IEAT was a combination of microorganisms resistant to ceftriaxone (27.2%) and microorganisms intrinsically non-susceptible to ceftriaxone, such as *Enterococcus* spp. or *Pseudomonas aeruginosa*. Modification of the initial empirical regimen showed a tendency in the group treated with ceftriaxone (52.5% vs. 39.3%) but was not statistically significant (*p* = 0.057). However, antibiotic escalation was significantly more frequent in the group of patients treated with ceftriaxone (43.2% vs. 24.8%, *p* = 0.045, OR 1.3 (CI 95% 1.1–1.6)). Despite that, no differences were observed in de-escalation between the two groups (Table 1). The most common escalation in the ceftriaxone group was to meropenem (70.6%), followed by piperacillin–tazobactam (9.8%). Escalation was not related to 30-day mortality (*p* = 0.815, OR 1.1 (CI 95% 0.6–2.1)), but it showed a longer hospital stay (6 [4–8] vs. 4 [3–6] days, *p* = 0.026).

**Figure 2:**
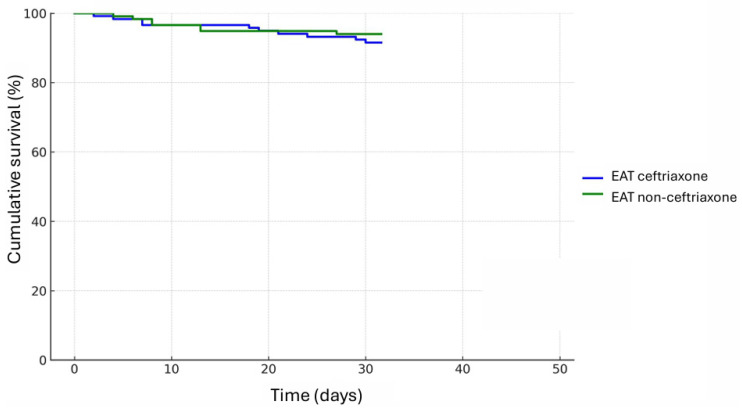
Thirty-day mortality among patients treated with ceftriaxone versus other antimicrobials as empirical antibiotic therapy (EAT) for community-onset healthcare associated urinary tract infection.

In-hospital mortality was 5.5% and 30-day mortality was 11.1%, with no significant differences between the ceftriaxone and non-ceftriaxone groups (5.1% vs. 6%, *p* = 0.763, and 11.9% vs. 10.3%, *p* = 0.694, respectively). Hazard ratio adjusted by age showed similar results (HR 1.11 (95% CI 0.63–1.98)), as well as a Kaplan–Meier analysis (Figure 2). Age < 80 years was confirmed as an independent mortality factor (HR 0.14 (95% CI 0.07–0.38) in the adjusted Cox model).

However, the use of antibiotics other than ceftriaxone as empirical therapy was associated with a longer hospital stay (6 [4–8] vs. 5 [3–7] days, *p* = 0.040). The use of ceftriaxone as empirical therapy was not associated with a higher risk of recurrence (16.1% vs. 15.4%, *p* = 0.880).

The prognostic variables mortality at 30 days after discharge, readmission due to recidivate UTI and length of stay were analyzed in more detail using multivariate analysis by logistic regression and negative-binomial regression (Table 4). In different multivariate analysis, only qSOFA and SOFA were independently related to 30-day mortality. In the multivariate analysis for readmission, severe dependency (Barthel ≤ 40) almost tripled the risk of readmission. Regarding longer hospital stay, in the univariate analysis Charlson ≥ 3, age ≥ 80 years, Barthel ≤ 40, dementia, qSOFA ≥ 2, SOFA ≥ 2, change of antimicrobials in the first 24–48 h and ESBL-Enterobacterales were related. The multivariate analysis using negative binomial regression revealed that empirical treatment with ceftriaxone was associated with a reduced length of stay (*p* = 0.037; IRR [expβ] = 0.87; 95% CI, 0.67–0.94). In contrast, a qSOFA score ≥ 2 showed a trend toward a longer hospital stay (IRR [expβ] = 1.27; 95% CI, 0.90–1.78), although this association was not statistically significant.

A sub-analysis of patients with sepsis, considering IEAT, was conducted for 30-day mortality. Fourteen (36.8%) cases had IEAT in the patients with sepsis treated with ceftriaxone, 4 of them died. Ten (30.3%) of the septic patients treated with other antibiotics had IEAT; with 1 case of mortality at 30 days. The differences between groups were not statistically significative.

## 4. Discussion

In this study, the empirical use of ceftriaxone in patients with community-onset healthcare-associated UTI was associated with a shorter hospital stay, without an increased risk of mortality or recurrence. However, the rate of inappropriate empirical therapy was higher in the ceftriaxone group.

Ceftriaxone is one of the most frequently used empirical therapies for UTI [3]. However, according to the 2025 Infectious Diseases Society of America (IDSA) guidelines, its use should be avoided in patients with known risk factors for resistance or in settings where local resistance rates exceed 10–20% in severe presentations [17]. A major concern is the increasing prevalence of ESBL-producing Enterobacterales, against which ceftriaxone is ineffective [17]. Importantly, emergency-department studies specifically highlight that when third-generation-cephalosporin resistance is present, discordant initial therapy is common in febrile UTIs [18]. Furthermore, multicenter cohorts have documented substantial resistance to third-generation cephalosporins and fluoroquinolones in hospitalized complicated UTIs, with multidrug resistance linked to carbapenem use, longer length of stay, and higher costs [5].

However, evidence on the impact of inadequate empirical therapy in UTIs is inconsistent. Some studies associate discordant therapy with worse outcomes, such as treatment failure or prolonged hospitalization in bacteremic patients [19]. Nevertheless, others found no significant differences, particularly those including clinically stable patients [20,21]. Recent evidence also suggests that using agents with high community resistance rates may be safe in stable patients when early reassessment and prompt therapy adjustment are ensured, highlighting the importance of timely clinical review and stewardship-led optimization [11].

In our study, the ceftriaxone group had a higher rate of IEAT (36.4% vs. 17.1%, *p* = 0.001), consistent with previous findings. For instance, Kayaaslan et al. [22] reported that treatment inappropriateness was most frequently associated with the empirical use of ceftriaxone (56.3%) in patients with uncomplicated pyelonephritis, with IEAT rates similar to those observed in our cohort.

In our study, inappropriate empirical therapy was not associated with increased in-hospital or 30-day mortality. Similar findings have been reported in other cohorts. For example, Nocua-Báez et al. [23] observed no differences in in-hospital mortality when comparing empirical cefazolin with other regimens in patients with community-acquired acute pyelonephritis due to Enterobacterales (OR 1.02, 95% CI 0.47–2.19). Likewise, in a multinational, multicenter retrospective cohort of hospitalized patients with complicated UTI, which included those with HCA-UTI, Eliakim et al. [21] found that IEAT was not associated with 30-day mortality, which was instead linked to factors such as septic shock, older age, and urinary catheter use. These findings are consistent with and support our results, in which 30-day mortality was associated with factors related to infection severity and patient age, but not with IEAT or the empirical use of ceftriaxone.

Escalation and de-escalation of empirical antibiotics should be conducted on an individual basis, assessing the patient’s clinical evolution, the patient’s own clinical characteristics, risk factors for resistance, and the results of the microbiological tests performed [17]. In a Spanish cohort of patients with healthcare-associated pyelonephritis initially treated with ceftriaxone, Bosch et al. [24] reported escalation to broader-spectrum antibiotics in approximately 40% of cases, most often due to ESBL-producing microorganisms, findings consistent with ours (34% overall escalation). The SIMPLIFY trial [25] demonstrated that structured de-escalation from an antipseudomonal β-lactam to a narrower-spectrum antibiotic, which included ceftriaxone, based on susceptibility, in patients with Enterobacteriaceae bacteremia (38% urinary tract infection) was non-inferior to continued empirical treatment in terms of clinical cure rate, mortality, and adverse events. In contrast, modifications of empirical therapy based on clinical response have been less frequently addressed. In a Taiwanese study of Enterobacterales bacteremia, Lee et al. [26] observed escalation in only 10.1% of cases, while de-escalation—more common in their cohort—was feasible in 50.9% of patients, in contrast to the results of our study, in which de-escalation in the first 24–48 h was only 11.9%, with no differences between groups. More studies on the clinical impact of escalation and de-escalation in patients with urinary tract infection would be interesting.

The use of ceftriaxone was associated with a shorter hospital stay in our study (5 vs. 6 days, *p* = 0.040). Similar findings have been reported by Zilberberg and Nocua-Báez [5,23]. In contrast, other studies have described a longer hospital stay [27,28] or no significant differences [29].

A study in patients with community-acquired acute pyelonephritis due to Enterobacterales reported an increased risk of recurrence with the use of cefazolin as empirical therapy (OR 3.7, 95% CI 2.4–5.8) [23]. In contrast, we found no significant differences in recurrence rates between our study groups, in line with the findings of Greenhouse and Zilberberg [5,20]. In the analysis by Greenhouse et al. [20] of different antimicrobial regimens for UTI caused by ESBL-producing Enterobacterales, inappropriate empirical therapy was not associated with increased re-hospitalization for ESBL-related UTI (18% vs. 14% in the inappropriate vs. appropriate groups, *p* = 0.858), results similar to ours. Conversely, in a large retrospective cohort of 23.331 patients with complicated UTI, Zilberberg et al. [5] observed that inappropriate therapy was associated with an increased risk of recurrence.

Ceftriaxone resistance in HCA-UTI was 29.8% in our cohort, similar in populations alike, such a Spanish study by Bosch et al. [24], in HCA acute pyelonephritis (32.6%), but higher than others in different settings, like a study in the Emergency Department in the USA [18], in which third generation cephalosporin resistance was only 12.9%. On the other hand, resistance rates to ciprofloxacin (46.6%) and cotrimoxazole (44.1%) in our study were lower compared to that in a 2017 Spanish study by Bosch et al. [24], in which resistance rates were 74.5% to ciprofloxacin and 58.7% to trimethoprim/sulfamethoxazole. This fact is probably related to the reduction in resistance to quinolones and trimethoprim/sulfamethoxazole observed in Europe between 2016 and 2023 in Gram-negative bacteria [30,31,32], in relation to a decrease in the use of these antibiotics [32]. Vancomycin resistance in Enterococcus spp. is low, consistent with what has been described in other European studies [33,34]. The case in our study was an *Enterococcus faecium* in a 74 years old man with prior use of antibiotics, previous hospitalization and use of urinary catheter. Notably, resistance to fosfomycin remains relatively low (10.8% in the Bosch study and 13.9% in ours) despite its widespread use in UTI, supporting its role as a valuable option for stable patients “Supplementary Table S1”.

Accepted practice in many European settings nevertheless varies considerably. A survey comparing national guidelines across 15 countries showed wide heterogeneity in first-line recommendations for UTIs, including differences in preferred parenteral agents and the treatment of suspected ESBL risk [35]. This diversity likely reflects the tension between avoiding IEAT and minimizing unnecessary broad-spectrum coverage, particularly carbapenems, in an era of rising resistance. Contemporary guidance emphasizes pragmatic principles: applying local antibiograms, identifying individual ESBL/Pseudomonas risk factors, using the narrowest appropriate empirical regimen, and adjusting rapidly based on culture results and clinical evolution [17,35]. The key issue is not whether ceftriaxone should be avoided universally, but in which patients and contexts it remains an appropriate initial choice.

In this context, our study provides real-world evidence in community-onset HCA-UTI. Although empirical ceftriaxone was associated with higher IEAT, it was not linked to greater 30-day mortality or recurrence and was instead associated with a shorter hospital stay. These results align with previous studies indicating that age and severity are the main mortality drivers, while the negative impact of discordant therapy can be offset by early recognition, prompt adjustment, and structured stewardship. Therefore, in selected patients without clear ESBL/Pseudomonas risk, and within systems ensuring rapid reassessment, ceftriaxone remains a reasonable empirical option.

One of the main limitations of this study is that decisions regarding antibiotic therapy were made at the discretion of the treating physicians, which may have introduced selection bias. However, only dementia and urinary catheter use differed slightly between the ceftriaxone and alternative empirical regimen groups among all clinic-epidemiological variables analyzed at admission. Even so, we cannot rule out that prescribing decisions may have been influenced by unrecorded factors such as prior urine culture results, previous antimicrobial exposure, or adverse drug reactions. This lack of standardization introduces the potential for confounding and limits the ability to draw causal inferences. Nonetheless, the study reflects the use of ceftriaxone under real-world clinical conditions, thereby enhancing the external validity of our findings and supporting their applicability to everyday clinical practice.

## 5. Conclusions

In summary, empirical ceftriaxone use in patients with community-onset healthcare-associated UTI was associated with a higher rate of inappropriate empirical therapy; however, it did not appear to increase mortality or recurrence, and it was associated with a shorter length of stay. We cannot recommend the empirical use of ceftriaxone for septic shock, as these patients were not included in the study; nonetheless, among the remaining population, ceftriaxone may represent a reasonable empirical option when guided by local resistance patterns and individual risk factors.

## Figures and Tables

**Table 1:** Epidemiological and clinical characteristics of patients with community-onset healthcare associated-UTIs treated empirically with ceftriaxone monotherapy versus other antibiotics.

	Total *N* 235	EAT Ceftriaxone *N* 118 (50.2)	EAT Non-Ceftriaone *N* 117 (49.8)	*p*
Female sex, *n* (%)	111 (47.2)	60 (50.8)	51 (43.6)	0.265
Age (years), median [IQR]	79 [72–86]	80 [73–88]	79 [72–85]	0.149
Charlson Index ≥ 3, *n* (%)	217 (92.3)	107 (90.7)	110 (94)	0.336
Barthel < 40, *n* (%)	95 (40.4)	52 (44.1)	43 (36.8)	0.253
**Comorbidities**				
Dementia, *n* (%)	79 (33.6)	48 (40.7)	31 (26.5)	**0.021**
Diabetes mellitus, *n* (%)	80 (34)	42 (35.6)	38 (32.5)	0.614
COPD, *n* (%)	42 (17.9)	21 (17.8)	21 (18.1)	0.951
CKD, *n* (%)	77 (33)	40 (34.2)	37 (31.9)	0.710
Cancer, *n* (%)	57 (24.3)	27 (22.9)	30 (25.6)	0.622
**Clinical characteristics**				
APACHE II, median [IQR]	11 [8–15]	11 [7–14]	11 [8–15]	0.258
APN, *n* (%)	161 (68.5)	79 (66.9)	82 (70.1)	0.605
Altered mental status, *n* (%)	95 (40.4)	45 (38.1)	50 (42.7)	0.473
RR ≥ 22 bpm, *n* (%)	41 (17.4)	19 (16.1)	22 (18.8)	0.585
qSOFA ≥ 2, *n* (%)	44 (18.7)	20 (16.9)	24 (20.5)	0.484
Sepsis (SOFA ≥ 2), *n* (%)	84 (35.7)	38 (32.2)	46 (39.3)	0.255
Urinary catheter, *n* (%)	65 (27.7)	29 (24.6)	36 (30.8)	0.289
Albumin, median [IQR]	3.3 [3–3.6]	3.3 [3–3.6]	3.3 [2.9–3.7]	0.787
White blood cell count × 10^9^/L, median [IQR]	12,850 [8900–17,075]	13,000 [9100–17,600]	12,500 [8600–16,000]	0.455
Bacteremia: Positive BC/Total BC (%)	47/128 (36.7)	20/56 (35.7)	27/72 (37.5)	0.982
Polymicrobial UTI, n (%)	17 (7.2)	6 (5.1)	11 (9.4)	0.201
IEAT, n (%)	63 (26.8)	43 (36.4)	20 (17.1)	**0.001**
Antimicrobial changes in the first 24–48 h, n (%)	108 (45.9)	62 (52.5)	46 (39.3)	0.057
Scalation	80 (34)	51 (43.2)	29 (24.8)	**0.045**
De-escalation	28 (11.9)	11 (9.3)	17 (14.5)	0.303
No changes	127 (54)	56 (47.5)	71 (60.7)	0.057

EAT, empirical antimicrobial therapy; COPD, chronic obstructive pulmonary disease; CKD, chronic kidney disease; APN, acute pyelonephritis; RR, respiratory rate; BC, blood culture; IEAT, inadequate empirical antimicrobial therapy. *p* < 0.05 is considered statistically significant (in bold).

**Table 2:** Microorganisms identified in community-onset healthcare associated-UTIs treated empirically with ceftriaxone monotherapy versus other antibiotics.

	Total *N* 253	EAT Ceftriaxone *N* 124 (49.1)	EAT Non-Ceftriaxone *N* 129 (50.9)	*p*
**Gram-negative bacteria**				
*Escherichia coli*	133(52.6)	70 (56.5)	63 (48.8)	0.277
*Klebsiella pneumoniae*	36 (14.2)	17 (13.7)	19 (14.7)	0.959
*Klebsiella oxytoca*	8 (3.2)	4 (3.2)	4 (3.1)	0.762
*Proteus mirabilis*	6 (2.4)	3 (2.4)	3 (2.3)	0.716
*Pseudomonas aeruginosa*	23 (9.1)	8 (6.5)	15 (11.6)	0.225
Other Gram-negative bacteria	22 (8.7)	9 (7.3)	13 (10.1)	0.567
**Gram-positive bacteria**				
*Enterococcus faecalis*	20 (7.9)	10 (8.1)	10 (7.8)	0.888
*Enterococcus faecium*	3 (1.2)	2 (1.6)	1 (0.7)	0.973
*Enterococcus gallinarum*	2 (0.8)	1 (0.8)	1 (0.7)	0.495

EAT, Empirical Antibiotic Therapy.

**Table 3:** Resistance rates in community-onset healthcare associated-UTI isolates: ceftriaxone monotherapy versus other empirical regimens.

	Total *N* 253	EAT Ceftriaxone *N* 124	EAT Non-Ceftriaxone N 129	*p*
MDRB, *N* (%)	108 (42.7)	49 (39.5)	59 (45.7)	0.171
ESBL-E, *N* (%)	45 (17.8)	20 (16.1)	25 (19.4)	0.389
Resistant/tested (%)				
Ampicillin	185/235 (78.7)	91/118 (77.12)	94/117 (80.34)	0.657
Amoxicillin-clavulanate	43/210 (20.5)	17/105 (16.2)	26/105 (24.8)	0.171
Piperacillin-tazobactam	3/225 (1.3)	0/111 (0)	3/114 (2.6)	0.255
Cephazolin	77/204 (37.7)	31/103 (30.1)	46/101 (45.6)	0.053
Ceftriaxone	61/205 (29.8)	28/103 (27.2)	33/102 (32.4)	0.512
Ceftazidime	52/227 (22.9)	24/111 (21.6)	28/116 (24.1)	0.769
Ertapenem	5/204 (2.5)	2/102 (1.9)	3/102 (2.9)	1
Meropenem	4/225 (1.8)	1/110 (0.9)	3/115 (2.6)	0.646
Gentamicin	57/252 (22.6)	26/124 (20.9)	31/128 (24.2)	0.641
Ciprofloxacin	118/253 (46.6)	57/124 (45.9)	61/129 (47.3)	0.933
Trimethoprim/sulfamethoxazole	101/229 (44.1)	40/112 (35.7)	61/117 (52.1)	0.057
Fosfomycin	30/219 (13.7)	13/109 (11.9)	17/110 (15.5)	0.573
Vancomycin	1/33 (3)	0/14 (0)	1/19 (5.3)	0.876

EAT, empirical antimicrobial therapy; MDRB, multidrug-resistant bacteria; ESBLs, extended spectrum beta-lactamase-producing *Enterobacterales.*

**Table 4:** Multivariate analysis of 30-day mortality, readmission, and length of stay in patients with community-onset healthcare associated-UTI.

	Univariate *p*	Multivariate *p*	OR (95% CI)	AUC (95% CI)
30-day mortality *				
Ceftriaxone	0.694	0.752	1.17 (0.49–3.04)	0.480 (0.362–0.597)
IEAT	0.923	0.898	0.93 (0.33–2.68)	0.504 (0.396–0.612)
Age ≥ 80 years	0.001	0.109	2.52 (0.82–7.77)	0.341 (0.245–0.437)
Barthel ≤ 40	<0.001	0.146	2.33 (0.75–7.25)	0.332 (0.233–0.431)
Dementia	0.002	0.248	1.85 (0.65–5.25)	0.359 (0.252–0.466)
Diabetes	0.013	0.066	2.35 (0.94–5.87)	0.388 (0.280–0.495)
qSOFA ≥ 2	<0.001	0.001	4.92 (1.86–12.98)	0.717 (0.622–0.813)
SOFA ≥ 2	<0.001	-	-	-
Readmission *				
Ceftriaxone	0.880	0.709	1.15 (0.55–2.43)	0.493 (0.392–0.595)
IEAT	0.733	0.249	0.59 (0.24–1.44)	0.496 (0.388–0.604)
Barthel ≤ 40	0.023	0.007	2.84 (1.33–6.04)	0.668 (0.569–0.767)
CKD	0.047	0.059	0.44 (0.18–1.03)	0.535 (0.424–0.647)
Urinary catheter	0.042	0.068	2.06 (0.95–4.47)	0.464 (0.359–0.568)
Length of Stay **	Univariate *p*	Multivariate *p*	IRR (expβ) (95% CI)	
Ceftriaxone	0.019	0.037	0.87 (0.67–0.94)	
IEAT	0.141	0.187	1.23 (0.90–1.67)	
Charlson ≥ 3	0.008	0.219	1.32 (0.85–2.06)	
Age ≥ 80 years	0.039	0.759	1.06 (0.75–1.50)	
Barthel ≤ 40	0.011	0.040	1.37 (1.01–1.86)	
Dementia	0.039	-	-	
qSOFA ≥ 2	0.002	0.169	1.27 (0.90–1.78)	
SOFA ≥ 2	<0.001	-	-	
ESBL-*Enterobacterales*	0.049	0.409	1.14 (0.83–1.54)	
Change of antimicrobials in the first 24–48 h	0.005	0.391	1.12 (0.86–1.45)	

IEAT, inadequate empirical antimicrobial therapy; CKD, chronic kidney disease; ESBL-E, extended spectrum beta-lactamase-producing *Enterobacterales.* * 30-day mortality and readmission multivariate analysis was done by logistic regression; ** Length of Stay multivariate analysis was conducted by negative-binomial regression.

## Data Availability

The original contributions presented in the study are included in the article, further inquiries can be directed to the corresponding author.

## Supplementary Materials

The following supporting information can be downloaded at: https://www.mdpi.com/article/10.3390/jcm14248761/s1, Table S1: Resistance rates in community-onset healthcare-associated UTI isolates: ceftriaxone monotherapy versus other empirical regimens by microorganisms.
