# Supplementary material for: Clinical Impact of Empiric Ceftriaxone for Hospitalized Patients with Community-Onset Healthcare-Associated UTIs
Source: J Clin Med. 2025 Dec 11;14(24):8761. doi: 10.3390/jcm14248761 (PMC12734003; doi:10.3390/jcm14248761)
Supplement: Supplementary file 1 [file jcm-14-08761-s001.zip › jcm-3953163-supplementary.pdf]

Suppl Table. Resistance rates in community-onset healthcare-associated UTI isolates: ceftriaxone monotherapy versus other empirical regimens by microorganisms

	Total	EAT Ceftriaxone	EAT non-ceftriaxone	p
Escherichia coli, n	133	70	63	
ESBL, n (%)	33 (24.8)	16 (22.8)	17 (26.9)	0.727
Resistant/ tested (%)				
Ampicillin	105/131 (80.2)	55/69 (79.7)	50/62 (80.6)	0.932
Amoxicillin-clavulanate		7/70 (10)	6/63 (9.5)	0.841
Piperacillin-tazobactam	2/132 (1.4)	0/70	2/62 (3.2)	0.424
Cephazolin	45/133 (33.8)	22/70 (31.4)	23/63 (36.5)	0.664
Ceftriaxone	42/133 (31.6)	21/70 (30)	21/63 (33.3)	0.821
Ceftazidime	37/133 (27.8)	19/70 (27.1)	18/63 (28.6)	0.992
Ertapenem	2/133 (1.5)	2/70 (2.9)	0/63	0.523
Meropenem	1/132 (0.7)	1/69 (1.4)	0/63	0.964
Gentamicin	27/133 (20.3)	16/70 (22.9)	11/63 (17.4)	0.578
Ciprofloxacin	75/133 (56.4)	39/70 (55.7)	36/63 (57.1)	0.993
Trimethoprim/ sulfamethoxazole	58/133 (43.6)	24/70 (34.3)	34/63 (53.9)	**0.035**
Fosfomycin	9/132 (6.8)	3/69 (4.3)	6/63 (9.5)	0.405
Klebsiella pneumoniae, n	36	17	19	
ESBL, n (%)	9 (25)	2 (11.8)	7 (36.8)	0.177
Resistant/ tested (%)				
Amoxicillin-clavulanate	7/36 (19.4)	2/17 (11.8)	5/19 (26.3)	0.497
Piperacillin-tazobactam	0/36	0/17	1/19 (5.3)	0.955
Cephazolin	13/36 (36.1)	3/17 (17.6)	10/19 (52.6)	0.066
Ceftriaxone	10/36 (27.8)	3/17 (17.6)	7/19 (36.8)	0.362
Ceftazidime	9/36 (25)	2/17 (11.8)	7/19 (36.8)	0.177
Ertapenem	0/36	0/17	0/19	-
Meropenem	0/36	0/17	0/19	-
Gentamicin	8/36 (22.2)	2/17 (11.8)	6/19 (31.6)	0.305
Ciprofloxacin	9/36 (25)	3/17 (17.6)	6/19 (31.6)	0.563
Trimethoprim/ sulfamethoxazole	11/36 (30.6)	4/17 (23.5)	7/19 (36.8)	0.615
Fosfomycin	12/34 (35.3)	7/17 (41.2)	5/17 (29.4)	0.719
Pseudomonas aeruginosa, n	23	8	15	
Resistant/ tested (%)				
Piperacillin-tazobactam	0/23	0/8	0/15	-
Ceftazidime	2/23 (8.7)	1/8 (12.5)	1/15 (6.7)	0.761
Meropenem	1/23 (4.3)	0/8	1/15 (6.7)	0.744
Gentamicin	3/23 (13)	1/8 (12.5)	2/15 (13.3)	0.553
Ciprofloxacin	11/23 (47.8)	3/8 (37.5)	8/15 (53.3)	0.775

Enterococcus faecalis, n	20	10	10	
Resistant/ tested (%)				
Ampicillin	0/20	0/10	0/10	-
Ciprofloxacin	16/20 (80)	7/10 (70)	9/10 (90)	0.576
Fosfomycin	0/16	0/8	0/8	-
Vancomycin	0/20	0/10	0/10	-
Gentamicin Sinergy	11/20 (55)	5/10 (50)	6/10 (60)	1

EAT, Empirical Antibiotic Therapy; ESBL, Extended spectrum beta-lactamase
